# A three-gene signature might predict prognosis in patients with acute myeloid leukemia

**DOI:** 10.1042/BSR20193808

**Published:** 2020-06-03

**Authors:** Xin Zhu, Qian Zhao, Xiaoyu Su, Jinming Ke, Yunyun Yi, Jing Yi, Jiang Lin, Jun Qian, Zhaoqun Deng

**Affiliations:** 1 Department of Laboratory Center, The Affiliated People’s Hospital of Jiangsu University, Zhenjiang 212002, Jiangsu, People’s Republic of China; 2 The Key Lab of Precision Diagnosis and Treatment of Zhenjiang City, Zhenjiang, Jiangsu, People’s Republic of China; 3 Department of Hematology, The Affiliated People's Hospital of Jiangsu University, Zhenjiang 212002, Jiangsu, People’s Republic of China; 4 Faculty of Forestry, Zhejiang A&F University, Hangzhou 310020, Zhejiang, People's Republic of China

**Keywords:** Acute Myeloid Leukemia (AML), Biomarker, Gene Signature, Prognosis

## Abstract

The identification of effective signatures is crucial to predict the prognosis of acute myeloid leukemia (AML). The investigation aimed to identify a new signature for AML prognostic prediction by using the three-gene expression (octamer-binding transcription factor 4 (OCT4), POU domain type 5 transcription factor 1B (POU5F1B) and B-cell-specific Moloney murine leukemia virus integration site-1 pseudogene 1 (BMI1P1). The expressions of genes were obtained from our previous study. Only the specimens in which three genes were all expressed were included in this research. A three-gene signature was constructed by the multivariate Cox regression analyses to divide patients into high-risk and low-risk groups. Receiver operating characteristic (ROC) analysis of the three-gene signature (area under ROC curve (AUC) = 0.901, 95% CI: 0.821–0.981, *P*<0.001) indicated that it was a more valuable signature for distinguishing between patients and controls than any of the three genes. Moreover, white blood cells (WBCs, *P*=0.004), platelets (PLTs, *P*=0.017), percentage of blasts in bone marrow (BM) (*P*=0.011) and complete remission (CR, *P*=0.027) had significant differences between two groups. Furthermore, high-risk group had shorter leukemia-free survival (LFS) and overall survival (OS) than low-risk group (*P*=0.026; *P*=0.006), and the three-gene signature was a prognostic factor. Our three-gene signature for prognosis prediction in AML may serve as a prognostic biomarker.

## Introduction

Acute myeloid leukemia (AML) is a heterogeneous disorder characterized by infiltration of blood, bone marrow (BM) and other tissues by clonal, proliferative, abnormally differentiated and poor morphology, cytochemistry, immunophenotype, cytogenetics and molecular abnormalities of leukemia population. It is a highly heterogeneous disease that may be resulted from gene mutation or overexpression [1–3]. Despite 75–85% of patients can achieve complete remission (CR) after induction chemotherapy, the 5-year survival is still less than 50% [4]. The median survival of patients over 65 years old is less than 1 year, and only 20% of patients survive for more than 2 years [5]. In order to make better treatment decision, more effective signatures are needed [6]. Developments in molecular genetics, particularly in cytogenetic results and molecular abnormalities, stimulated the identification of prognostic signatures of AML [7].

Octamer-binding transcription factor 4 (OCT4), is considered as a putative cancer stem cells (CSCs) marker, which is a member of the Pit-Oct-Unc (POU) transcription factor family, mediating tumor proliferation and differentiation, abnormally expressed in bladder cancer, non-small cell lung cancer, cervical cancer and other cancers [8–13]. The previous study of our research group showed that overall survival (OS) of patients with OCT4 high expression is shorter than those with low expression, which suggested that high expression of OCT4 indicates unfavorable prognosis in AML [14].

Pseudogenes, which are derived from gene mutations, or unfaithful gene duplications, or retrotransposition of processed mRNAs back into the genome, neither in health nor in diseases, particularly in cancer, act on multiple levels (DNA, RNA and protein) significantly [15]. POU domain type 5 transcription factor 1B (POU5F1B) is a pseudogene that is highly homologous to OCT4. Recently it has been found to be transcribed in cancer cells. For example, in gastric cancer (GC), overexpressed POU5F1B was found to stimulate the occurrence and growth of tumors *in vivo*. Therefore, POU5F1B amplification is considered as a new prognostic factor for advanced GC patients [16]. According to our previous research, the expression of POU5F1B was down-regulated in AML compared with control, which might have unfavorable prognosis [17].

Polycomb group gene B-cell-specific Moloney murine leukemia virus integration site-1 (BMI1) is crucial to regulate the proliferative activity of normal and leukemia stem cells. Julie et al. demonstrated that leukemic stem cells and progenitor cells lacking BMI1 will suffer from proliferation stagnation, which will impair their proliferation potential and eventually lead to leukemia transplantation failure [18]. BMI 1 pseudogene 1 (BMI1P1), BMI1 pseudogene, is highly homologous to BMI1. Our earlier study indicated that BMI1P1 is frequently down-regulated in AML patients and low-expressed BMI1P1 of AML patients had obviously shorter leukemia-free survival (LFS) and OS than high-expressed [19].

The purpose of this investigation was to identify a new prognostic gene signature that is correlated with OS for AML prognostic prediction by using the three-gene expression (OCT4, POU5F1B and BMI1P1) data. More than that, we evaluated whether the new gene signature has a stronger prognostic value than any one of the three genes.

## Materials and methods

### Data sources

The data of gene expression were obtained from the preliminary research of our research group [14,17,19]. The data from the common specimens of the three genes were picked for further study, including 15 healthy donors as normal controls and 88 *de novo* AML patients who were diagnosed by the French–American–British (FAB) [20] and World Health Organzation (WHO) classifications [21]. The patients received the treatment according to previous reported standard [22] including induction therapy and subsequent consolidation treatment. For non-acute promyelocytic leukemia (non-APL) patients, induction therapy included one or two courses of daunorubicin combined with cytarabine. Subsequent consolidation therapy included high-dose cytarabine, mitoxantrone with cytarabine and homoharringtonine combined with cytarabine. For acute promyelocytic leukemia (APL) patients, induction therapy was oral all-*trans* retinoic acid (ATRA) together with daunorubicin in combination with cytarabine. Maintenance therapy was oral mercaptopurine, oral methotrexate and oral ATRA over 2 years.

### Construction of the prognostic gene signature

Kaplan–Meier analysis was performed for patients with gene expression and survival. Whether the value of gene expression can distinguish AML patients from normal people is evaluated by receiver operating characteristic (ROC) curve and area under ROC curve (AUC). According to the cut-off value from ROC, patients were divided into low- and high-expression groups. In the present study, univariate Cox proportional hazards regression analysis was adopted to evaluate survival. Taking OS as dependent variable, a multivariate Cox regression model was used to fit these three genes to measure their relative contribution to survival prediction. A prognostic risk score was established according to the linear combination of the expression level of these genes and the regression coefficient (β) obtained by multivariate Cox proportional hazard regression model of each gene. The prognostic risk scoring formula is expression of gene1 * β1 + expression of gene2 * β2 + … expression of gene n * βn [23,24]. The AML patients were divided into low- and high-risk groups by the cut-off value of the prognostic risk score from the ROC curve.

### Statistical analyses

Statistical analyses were performed using SPSS 20.0 software package (IBM Corp, Armonk, NY, U.S.A.) and GraphPad Prism 7.0. To compare quantitative data between two groups, Mann–Whitney U-test was applied. And Chi square test or Fisher exact test was applied to analyze the difference of categorical variables between two groups. The ROC curve was used to determine the predicted values of the parameters. In addition, the AUC and ROC curve are executed to estimate the differentiated ability of expression level between patients and controls.

The prognostic value of gene for OS and LFS was analyzed by Kaplan–Meier analysis. Univariate and multivariate analyses were based on the Cox proportional hazards regression model. We used univariate Cox proportional hazards regression analysis to evaluate the association between patients’ OS and the expression of each gene. The risk score of a patient was achieved by the sum of multiplying the expression levels of each gene by its corresponding regression coefficient. A *P*-value, which was less than 0.05 (two-tailed), was considered statistically significant.

## Results

### A predictive model of the three-gene signature

As we discovered from the previous data, patients in AML with higher BMI1P1 and POU5F1B expression had significantly better OS than lower expression. On the contrary, patients with high-expressed OCT4 showed shorter OS in AML. As shown in Table 1, the hazard ratio (HR) and 95% CI were used to assess the risk of death in the high-expression group relative to the low-expression group and were calculated by the univariate Cox proportional hazards regression model. The three genes were prognostic factors for OS, and they were also independent factors for OS according to multivariable Cox proportional hazard regression analyses. Based on these results, we built up a risk score combining the relative expression level of these three genes.

**Table 1:** Univariate and multivariate analyses of prognostic factors for OS in AML patients

Gene symbol	Univariate analyses		Multivariate analyses		
	HR (95%CI)	*P*-value	HR (95%CI)	*P*-value	Coefficient β
*OCT4*	2.010 (1.190–3.395)	0.009	2.259 (1.330–3.834)	0.003	0.815
*POU5F1B*	0.430 (0.204–0.907)	0.027	0.403 (0.185–0.834)	0.015	−0.909
*BMI1P1*	0.463 (0.220–0.974)	0.042	0.393 (0.190–0.854)	0.018	−0.933

**Figure 1:**
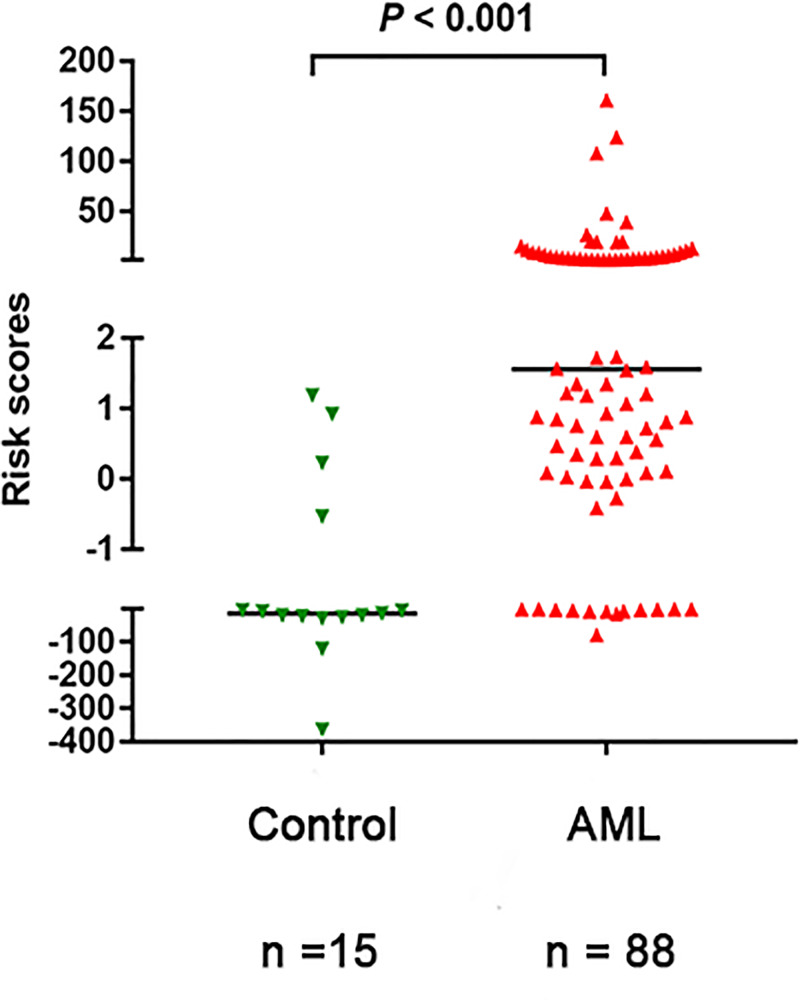
The risk scores of the three-gene signature in AML patients and controls Horizontal lines represent the median, and each dot represents an individual sample. Statistical analysis was performed using Mann–Whitney’s U test, and significance was defined as *P*<0.05.

The three-gene expression prognostic score of the predictive model was calculated as follows: 0.815 * OCT4 + (−0.933) * POU5F1B + (−0.909) * BMI1P1. The coefficients were calculated by Cox regression, and the gene name represents its expression level. Higher score indicated greater mortality risk for patient with AML. The risk score of the three genes collectively in AML (−79.060−161.220, median 1.557) detected higher compared with control (−364.020−1.190, median −13.972) (*P*<0.001, Figure 1).

### Comparison of ROC and AUC in three-gene signature and the three single genes

An ROC curve was constructed to analyze the diagnostic accuracy of gene expression. It revealed that OCT4, POU5F1B and BMI1P1 expression could serve as valuable biomarkers for distinguishing between AML and control subjects (AUC = 0.747, 95% CI: 0.627–0.867, *P*<0.001; AUC = 0.735, 95% CI: 0.587–0.883, *P*<0.01; AUC = 0.783, 95% CI: 0.624–0.941, *P*<0.001).

**Figure 2:**
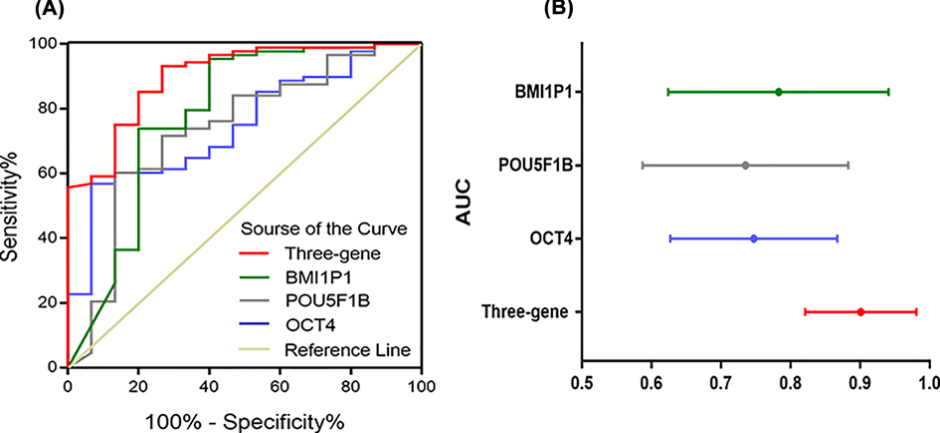
The predictive ability of the three-gene model was more robust The predictive ability of the three-gene signature compared with single markers by ROC and areas under the curve (AUC) (**A**) with 95% CI (**B**).

Compared with a single gene, in our current study the ROC curve of three-gene signature (AUC = 0.901, 95% CI: 0.821–0.981, *P*<0.001), confirming the prediction accuracy of this model, could serve as a more valuable signature for distinguishing between AML patients and control subjects. The results show that the predictive ability of the three-gene model was more robust than that any of the three genes (Figure 2).

### Clinical characteristics and prognostic significance of three-gene

This cohort of AML patients was divided into low-risk group and high-risk group by the cut-off value of 1.2 from the ROC curve of three-gene signature. As shown in Table 2, age, hemoglobin (Hb), 2016 WHO classification, Karyotype classification and gene mutations (C/EBPA, NPM1, FLT3 ITD, C-KIT, IDH1/2 and DNMT3A) did not differ significantly between low-risk group and high-risk group. However, the white blood cells (WBCs) and platelets (PLTs) in high-risk group were significantly higher than those in low-risk group (*P*=0.004; *P*=0.017). Also, the percentage of blasts in BM was significantly lower in low-risk group than high-risk group (*P*=0.011). In addition, distribution of karyotypes between two groups of AML patients had a significant difference (*P*=0.009). Furthermore, the result indicates that low-risk cases had remarkably higher CR than high-risk cases (*P*=0.027).

**Table 2:** Comparison of clinical and laboratory features in AML patients with low- and high risk groups

Patients parameters	Risk score group		
	Low-risk group (*n*=39)	High-risk group (*n*=49)	*P*-value
Sex, male/female	18/21	19/30	0.521
Median age, years (range)	54 (21–86)	55 (24–93)	0.551
Median WBC, ×10^9^/l (range)	7.8 (0.3–185.4)	41.4 (1.1–528)	0.004
Median Hb, g/l (range)	78.0 (34.0–131.0)	75.0 (42.0–138.0)	0.831
Median PLTs, ×10^9^/l (range)	39.5 (7.0–118.0)	52.0 (12.0–264.0)	0.017
BM blasts, % (range)	34.5 (1.0–90.0)	62.0 (3.0–97.7)	0.011
CR (+/−)	23/14	17/30	0.027
FAB			0.005
M0	0	0	
M1	1	6	
M2	16	16	
M3	14	6	
M4	4	17	
M5	3	4	
M6	1	0	
2016 WHO classification			0.100
AML with t(8;21) (q22;q22.1)	3	4	
AML with PML-RARA	14	6	
AML with mutated NPM1	4	3	
AML with biallelic mutations of CEBPA	2	0	
AML without maturation	0	4	
AML with maturation	9	11	
Acute myelomonocytic leukemia	4	17	
Acute monoblastic/monocytic leukemia	2	4	
Pure erythroid leukemia	1	0	
Karyotype classification			0.055
Favorable	17	10	
Intermediate	16	27	
Poor	3	10	
No data	3	2	
Karyotype			0.009
normal	15	16	
t(8;21)	3	4	
t(15;17)	14	6	
Others	3	11	
Complex	1	10	
No data	3	2	
Gene mutation			
*CEBPA (+/−)*	5/32	2/42	0.237
*NPM1 (+/−)*	4/33	3/41	0.551
*FLT3-ITD (+/−)*	3/34	7/37	0.332
*c-KIT (+/−)*	1/36	1/43	1.000
*IDH1/2 (+/−)*	1/36	2/42	1.000
*DNMT3A (+/−)*	2/35	5/39	0.445

**Figure 3:**
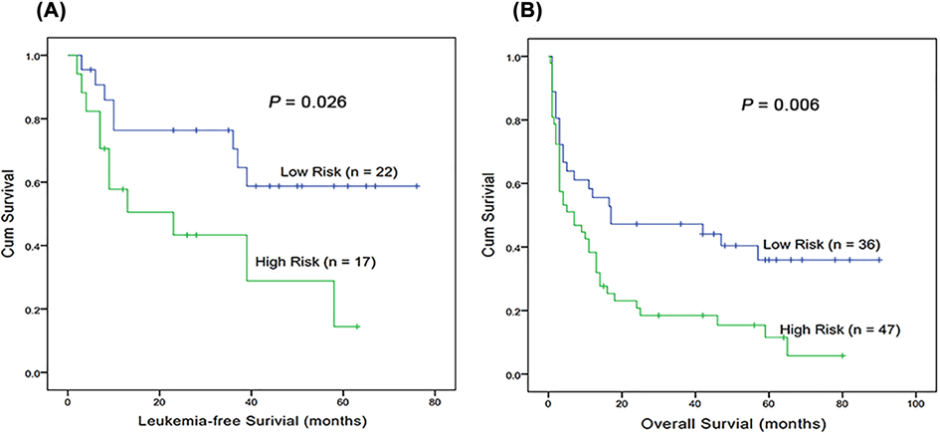
Low-risk group predicts favorable prognosis in AML (**A**) LFS was investigated for AML patients according to risk score of the three-gene signature. (**B**) OS was investigated for AML patients according to risk score of the three-gene signature.

LFS and OS were also assessed on the basis of Kaplan–Meier methods. Kaplan–Meier analysis demonstrated that patients of high-risk group had significantly shorter LFS (*P*=0.026, Figure 3A) and OS (*P*=0.006, Figure 3B) than that in the low-risk group. Univariate Cox proportional hazards regression analysis showed that the predictive model of a three-gene signature was a predictor for prognosis (HR = 2.003, 95% CI: 1.185–3.387, *P*=0.010).

## Discussion

Although gene expressions currently are not included in the standard diagnosis of AML, it is proved to be a comprehensive tool for leukemia diagnosis and classification due to its high accuracy in all clinically relevant leukemia subentity predictions [25,26]. Thus, a gene signature that can predict the prognosis of a large cohort of AML patients is of great significance. Because gene expression can capture the influence of the changes of multiple genes at the same time, a gene signature can summarize the prognosis of multiple ‘conventional’ risk markers into one score.

Recently, Sarah et al. identified a three-gene signature (CALCRL, CD109 and LSP1) predicts clinical outcomes’ prognostics in an AML research that accurately stratified survival, and exceeded the current ability of single molecule marker [6]. Additionally, Rui et al. successfully identified and verified an 11-gene signature for prognosis prediction of AML patients, which could well predict survival [27]. Furthermore, Montserrat et al. identified a four-gene expression prognostic signature might could be used to refine prognostic assessment and could guide postremission treatment in intermediate-risk cytogenetic AML patients [28].

In our study, the results indicated that the three-gene signature might be a prospective biomarker for distinguishing controls from AML. In addition, ROC analysis indicated that the predictive ability of the three-gene model was more robust than that any one of the three genes.

It was reported that AML patients with high WBC count have unfavorable prognostic [29]. It was also demonstrated that PLT count had a predictive value for the prognosis and survival of patients with AML patients [30]. Moreover, a higher PLT count was related to a higher CR rate [31]. Indeed, our study suggested that high-risk group of AML patients related with high WBC count and low PLT count had lower CR rate. In addition, patients of low-risk group had a better prognosis compared with patients with high-risk group. Importantly, our study further demonstrated that patients of low-risk group obtained significantly better LFS and OS in the AML cohort. This would be helpful for guiding for future therapy.

It will be interesting to explore how the genes in our signature are involved in the pathogenesis of the disease. We therefore compared our results with other literatures.

It has been demonstrated by experimental evidence that oncogenic growth in leukemias of both myeloid and lymphoid lineages is dependent on WNT signaling [32]. OCT4 is considered as a putative CSC marker and a key regulator of stem cell pluripotency and differentiation [33,34]. And overexpression of Oct4 may activate WNT signaling pathway to promote epithelial–mesenchymal transition (EMT), which in turn enhances the CSC-like properties and metastasis in hepatocellular carcinoma [35]. Thus, it can be assumed that up-regulation of OCT4 activates WNT signaling pathway and promotes oncogenic growth in AML. POU5F1B, which is also part of our predictor, is a pseudogene that is highly homologous to its parental gene OCT4. Pseudogenes are ideal candidates to sustain the expression of their parental genes by serving as competing endogenous RNAs (ceRNAs), which compete for the binding site of the same miRNAs [36,37]. Accordingly, POU5F1B may be functional by mediating miRNA expression in AML. Overexpression of POU5F1B may be expected to prevent the function of oncomiRs targeting essential genes to cellular repression, by competitive binding to the oncomiRs, and inhibit AML in some way. Another gene from our prognostic signature, BMI1P1, is a pseudogene of BMI1. BMI1 is a stem cell factor that is highly expressed in various human cancers, including AML [38,39]. Yu et al. found that overexpression of BMI1 activates the WNT pathway by inhibiting IDAX expression in colon cancer [40], so we further hypothesized that up-regulation of BMI1 activates the WNT pathway and leads to AML. And BMI1P1, which is similar to POU5F1B, is supposed to serve as ceRNAs to regulate expression of its parental coding genes BMI1.

The next step in our study is to design more experiments that include *in vitro* and *in vivo* functional assays, stem cell-related assays, and assays for the relationship between stem cell-related genes and their pseudogenes to assess the potential effects of stem cell-related genes and their pseudogenes on AML mechanism.

We also need to acknowledge that there are several limitations in our research. One limitation of the present study is the small sample size for analysis. Therefore, a large number of samples are needed to further validate the prognostic value of this three-gene signature on AML patients. Besides, the robustness of the three-gene signature need further proving in multiple, independent and prospective validation cohorts, especially in diverse populations of patients. However, at present, we have not discovered that the three genes exist any same public repository database, so it is recently impossible to further validate the three-gene expression risk score of prognostic value in database. Moreover, we could use high-throughput methods to screen gene combinations with higher specificity and sensitivity. Despite these limitations, our findings showed that the three-gene signature may have clinical utility for prognosis prediction in AML patients.

In conclusion, the three-gene signature we constructed for prognosis prediction in patients with AML may serve as a potential prognostic biomarker. Futhermore, the three-gene prognostic signature is strongly associated with the clinical outcome in AML patients and may have potential for clinical use in the future.

## Abbreviations

AML: acute myeloid leukemia
APL: acute promyelocytic leukemia
ATRA: all-*trans* retinoic acid
AUC: area under receiver operating characteristic curve
BM: bone marrow
BMI1: B-cell-specific Moloney murine leukemia virus integration site-1
BMI1P1: BMI1 pseudogene 1
ceRNA: competing endogenous RNA
CI: confidence interval
CR: complete remission
CSC: cancer stem cell
GC: gastric cancer
HR: hazard ratio
IDAX: inhibition of the Dvl and Axin complex
LFS: leukemia-free survival
OCT4: octamer-binding transcription factor 4
OS: overall survival
PLT: platelet
POU: Pit-Oct-Unc
POU5F1B: POU domain type 5 transcription factor 1B
ROC: receiver operating characteristic
WBC: white blood cell
WHO: World Health Organzation
WNT: Wingless and INT-1
